# Targeting miRNA with flavonoids: unlocking novel pathways in cardiovascular disease management

**DOI:** 10.3389/fphar.2025.1532986

**Published:** 2025-03-06

**Authors:** Arya Tjipta Prananda, Princella Halim, Rony Abdi Syahputra

**Affiliations:** ^1^ Faculty of Medicine, Universitas Sumatera Utara, Medan, Indonesia; ^2^ Department of Pharmacology, Faculty of Pharmacy, Universitas Sumatera Utara, Medan, Indonesia

**Keywords:** cardiovascular disease (CVD), microRNAs (miRNAs), flavonoids, therapeutic modulation, cardioprotection

## Abstract

Cardiovascular disease (CVD) remains the leading cause of mortality worldwide, with complex pathophysiological mechanisms such as oxidative stress, inflammation, apoptosis, and endothelial dysfunction driving disease progression. MicroRNAs (miRNAs), a class of non-coding RNAs, have emerged as key regulators of gene expression involved in these processes, positioning them as potential biomarkers and therapeutic targets in CVD management. Simultaneously, flavonoids, naturally occurring polyphenolic compounds found in various plant-based foods, have gained attention for their cardioprotective properties, including antioxidant, anti-inflammatory, and anti-apoptotic effects. Recent studies suggest a novel intersection between flavonoids and miRNAs, where flavonoids may modulate the expression of specific miRNAs implicated in CVD pathogenesis. This review explores the potential of flavonoids as miRNA modulators, focusing on their ability to regulate miRNAs associated with cardiac fibrosis, hypertrophy, and vascular inflammation. By bridging the therapeutic potential of flavonoids with miRNA targeting, this review highlights innovative pathways for advancing CVD treatment strategies. Additionally, preclinical and clinical evidence supporting these interactions is discussed, alongside the challenges and opportunities in developing flavonoid-based miRNA therapies. Unlocking this synergy could pave the way for more effective, personalized approaches to CVD management, addressing unmet needs in contemporary cardiovascular care.

## 1 Introduction

Cardiovascular disease (CVD) encompasses a range of heart and blood vessel disorders that are the leading cause of death worldwide. According to recent data, more than 17 million deaths each year are caused by CVD, with a significant upward trend in developing countries due to lifestyle changes, urbanization, and unhealthy diets. It encompasses a wide range of conditions such as coronary heart disease, hypertension, arrhythmias, heart failure, stroke, and peripheral vascular disease (Roth et al., 2019). The long-term impact of CVD is not only limited to individual health but also poses a heavy economic burden to society and the global health system (Powell-Wiley et al., 2022). The progression of CVD involves a complex set of mechanisms acting at the cellular and molecular levels. These mechanisms include oxidative stress, chronic inflammation, cellular apoptosis, endothelial dysfunction, as well as fibrosis which all contribute to cardiac and vascular remodeling (Masenga et al., 2023). A deep understanding of the molecular factors that influence the course of these diseases is essential for developing more effective prevention and treatment strategies. MicroRNAs (miRNAs) are a small class of non-coding RNAs that play a central role in the post-transcriptional regulation of various genes involved in biological processes (O’Brien et al., 2018). By inhibiting translation or facilitating degradation of target mRNAs, miRNAs regulate the expression of genes involved in the control of cellular functions, including cell growth, differentiation, apoptosis, and immune response (Seyhan, 2024). In the context of the cardiovascular system, miRNAs have been shown to be involved in a variety of important pathways that influence the pathophysiology of heart and vascular diseases (Condorelli et al., 2014). A number of miRNAs have been identified as key drivers in the development and progression of cardiovascular diseases. For example, miR-21 is known to play a role in cardiac fibrosis, miR-133 is associated with cardiac hypertrophy, and miR-155 plays an important role in vascular inflammatory responses. Dysregulation of the expression of these miRNAs not only triggers cardiac and vascular dysfunction but also exacerbates disease progression (Schoettler et al., 2024). As a result, miRNAs have been recognized as potential biomarkers for CVD diagnosis and prognosis as well as promising therapeutic targets (Chakrabortty et al., 2023). Recent research has focused on miRNA modification strategies to address various aspects of CVD, including the use of antagomiRs (miRNA antagonists) to inhibit the function of specific miRNAs or miRNA mimics to increase the expression of protective miRNAs (Peters et al., 2020). While this approach shows great potential, challenges in the delivery of therapeutic miRNAs as well as possible side effects require further research (Rupaimoole et al., 2011). Flavonoids are a group of polyphenolic compounds widely found in plant foods such as fruits, vegetables, tea, cocoa, and wine (Panche et al., 2016). Flavonoids are well known for their extensive biological properties, including as antioxidants, anti-inflammatory, anti-apoptotic, and anticancer agents (Ullah et al., 2020). Structurally, flavonoids can be classified into several subgroups, including flavonols, flavones, flavanones, isoflavones, and anthocyanins, each with different biological activity profiles (Chen et al., 2023). In the cardiovascular context, flavonoids have great potential to prevent and reduce the progression of CVD (Fusi et al., 2020). They have been shown to protect the endothelium from dysfunction resulting from oxidative stress and inflammation, two major factors in the development of atherosclerosis (Scioli et al., 2020). In addition, flavonoids are also able to promote vasodilation, reduce vascular inflammation, inhibit platelet aggregation, and improve lipid profiles, all of which contribute to the prevention of coronary heart disease and hypertension. Recent studies have shown that regular consumption of flavonoids is associated with a reduced risk of various types of cardiovascular diseases, including heart attack and stroke (Syahputra et al., 2024). Interestingly, several studies are now focusing on how flavonoids can modulate the expression of miRNAs involved in CVD pathogenesis. These interactions may open up new opportunities for the development of flavonoid-based molecular therapies that are more specific and effective in addressing cardiovascular dysfunction (Milenkovic et al., 2013). This review aims to explore the potential of flavonoids as miRNA modulators in the context of cardiovascular disease. On the one hand, flavonoids have been recognized as cardioprotective agents that work through various mechanisms, including the reduction of oxidative stress, inflammation, and apoptosis (Seyhan, 2024). On the other hand, miRNAs have become very attractive potential targets for regulating molecular pathways involved in CVD. Thus, the interaction between flavonoids and miRNAs offers a new perspective that may lead to the development of more advanced and personalized therapeutic strategies in the management of CVD. By utilizing the ability of flavonoids to modulate the expression of cardiovascularly relevant miRNAs, it is hoped that there will be new pathways that can be utilized for the treatment of these diseases. This review will also discuss the mechanisms by which flavonoids interact with miRNAs, as well as the preclinical and clinical evidence supporting the therapeutic effects of this modulation. Ultimately, we will underline the importance of further research in this area to fully understand the potential use of flavonoids in more effective miRNA-based treatments, as well as the challenges and opportunities that may arise.

## 2 MicroRNA: key regulator in cardiovascular pathophysiology

### 2.1 Overview of miRNA biogenesis and function

MicroRNAs (miRNAs) are a class of small non-coding RNAs that play a crucial role in post-transcriptional regulation of gene expression, and since their discovery in 1993, miRNAs have been identified as key regulators of many biological processes, including heart and vascular function (O’Brien et al., 2018; Bhaskaran and Mohan, 2014). The miRNA biogenesis process begins in the nucleus with the transcription of pri-miRNA by RNA polymerase II, which is then processed by the Drosha-DGCR8 complex into stalk-pinch structured pre-miRNA (Davis-Dusenbery and Hata, 2010). After being exported to the cytoplasm via exportin-5, the pre-miRNA undergoes further processing by Dicer, resulting in a miRNA duplex. One strand of this duplex, known as the guide strand, is incorporated into the RNA-induced silencing complex (RISC), where the miRNA binds to the target mRNA based on partial or full complementarity, resulting in translational inhibition or degradation of the mRNA (O’Brien et al., 2018). In the cardiovascular context, miRNAs play complex regulatory roles, particularly in maintaining cardiac and vascular homeostasis and regulating responses to pathological stress. Important pathways regulated by miRNAs include cell proliferation, migration, angiogenesis, hypertrophy and apoptosis, all of which are critical processes in maintaining healthy heart function (Zapata-Martínez et al., 2023). Changes in miRNA expression, even in small amounts, can disrupt this homeostatic balance, causing or exacerbating cardiovascular pathologies. More than just regulators of individual genes, miRNAs have the potential to regulate vast networks of genes, allowing them to affect multiple pathways at once. Within cardiac tissue, miRNA expression is cell-type specific, and many miRNAs are differentially expressed in the context of cardiovascular pathologies such as myocardial infarction, cardiac hypertrophy or atherosclerosis (Liu and Olson, 2010). Therefore, miRNAs are very attractive candidates as diagnostic and prognostic biomarkers, as well as therapeutic targets.

### 2.2 MiRNA dysregulation in cardiovascular disease

Cardiovascular disease (CVD) is characterized by dysfunction involving various molecular processes such as inflammation, apoptosis, fibrosis and endothelial dysfunction, all of which are strongly influenced by miRNA expression and regulation. Several specific miRNAs act as key regulators in cardiovascular pathways, and changes in the expression of these miRNAs can significantly influence disease progression (Table 1) (Kondracki et al., 2024).

**TABLE 1:** Several miRNA dysregulation.

miRNA	Subject	Target	Dysregulation	Dysregulation effects	References
miR-133a	Serum	FGFR1	Up	Early biomarker for stable CAD	Zhou et al. (2022)
miR-21, miR-208b	Plasma	TGF-β1/Smad-3 Signaling Pathway	Up	↑Cardiac fibrosis	Zhang et al. (2022b)
miR-29b (miR-let-7b)	Serum	Osteogenic transcription factors	Down	↑Coronary artery calcification incidence	Yang et al. (2022)
miR-203	Serum	Inflammatory cells	Up	Early biomarker for STEMI prediction	Li et al. (2022)
miR-125a (miR-125b, miR-223)	Plasma	Cardiomyocytes	Up	↑ACS Survival	Gager et al. (2022)
miR-132	Serum	PTEN, SIRT1	Down	Hypertension biomarker in obesity	Eikelis et al. (2022)
miR-21, miR-19a	Serum	Inflammatory cells	Up	Premature death risk from cancer and CVD	Yamada et al. (2021)
miR-126	Serum	Inflammatory cells	Down	Premature death risk from cancer and CVD	Yamada et al. (2021)
miR-222	Serum	PI3K/AKT pathway	Down	↑MI/R Incidence	Thottakara et al. (2021)
miR-1, miR-133a, miR-133b	*ND	Genes regulating ion channels	Up	Arrhythmia diagnostic biomarkers in pediatric patients	Moric-Janiszewska et al. (2021)
miR-1	Serum	Endothelial function, angiogenesis, apoptosis	Up	miR-1 within 3 h of acute chest pain is an independent mortality risk factor in MI patients	Su et al. (2020)
miR-155	Serum	Genes involved in pulmonary fibrosis and CAD	Down	↑miRNA on HF patients post-MI	Zhang et al. (2019)
miR-133a, miR-221	Plasma	HFrEF gene	Down	Elderly HF diagnostic biomarkers	Guo et al. (2018)

ACS, Acute Coronary Syndrome; CAD, Coronary Artery Disease; CVD, Cardiovascular disease; HF, Heart Failure; HFrEF, Heart Failure with Reduced Ejection Fraction; MI, Myocardial Infarction; STEMI, ST-Elevation Myocardial Infarction. *ND: the information is not provided by original article.

#### 2.2.1 miR-1

As one of the most abundant miRNAs in the heart, miR-1 plays a role in the regulation of cardiac contractility and electrophysiological homeostasis. Overexpression of miR-1 has been associated with an increased risk of arrhythmias, as miR-1 targets proteins that regulate ion channels, such as Kir2.1, which is important for cardiac rhythm stability. Dysregulation of miR-1 is also linked to myocardial infarction, where its decreased expression leads to cardiac contractile dysfunction (Yang et al., 2023; Benzoni et al., 2021). miR-133: Known as the “guardians of the heart,” miR-133 is instrumental in suppressing cardiac hypertrophy. In pathological hypertrophy conditions, miR-133 expression is drastically decreased, allowing activation of hypertrophic pathways such as the MEK-ERK1/2 Pathway (Li et al., 2018). In several studies in mouse models, upregulation of miR-133 was shown to reduce heart muscle cell size and improve cardiac function, making it a promising target for therapeutic intervention in hypertrophic conditions (Abdellatif, 2010). miR-21: miR-21 is one of the most significant miRNAs in the process of cardiac fibrosis. It plays a role by regulating the TGF-β pathway, which mediates fibroblast activation and extracellular matrix overproduction. Increased expression of miR-21 in the fibrotic heart increases cardiac fibroblast activity, leading to pathological fibrosis, tissue remodeling, and ultimately heart failure. Inhibition of miR-21 through antagomiRs has been shown to reduce cardiac fibrosis in animal models, showing great potential in the treatment of fibrotic heart conditions (Zhou X. L. et al., 2018; Chau et al., 2013). miR-126: Specific to endothelial cells, miR-126 plays a key role in maintaining vascular integrity and regulating angiogenesis through the VEGF (vascular endothelial growth factor) signaling pathway. Decreased expression of miR-126 has been associated with endothelial damage in patients with diabetes and other vascular diseases. Studies show that restoration of miR-126 can improve endothelial dysfunction and stimulate angiogenesis, especially in ischemia conditions (Sanguineti et al., 2021). Dysregulation of these and other miRNAs not only signals the presence and progression of cardiovascular disease, but also allows for more precise approaches in diagnosis and treatment, especially by identifying patterns of expression of specific miRNAs associated with specific phases of disease (Siasos et al., 2020).

### 2.3 miRNAs as therapeutic targets in cardiovascular disease

The ability of miRNAs to regulate the expression of hundreds of genes at a time makes them attractive therapeutic targets in the context of cardiovascular disease. Approaches that utilize miRNA modification, such as inhibition of pathogenic miRNAs or amplification of protective miRNAs, offer innovative therapeutic pathways and the potential to modify molecular pathways involved in the pathogenesis of cardiovascular disease (Ha, 2011).

#### 2.3.1 AntagomiRs

AntagomiRs are synthetic molecules designed to bind and inactivate specific miRNAs (Gustincich et al., 2016). For example, the use of antagomiRs to inhibit miR-21 has been shown to be effective in reducing cardiac fibrosis in heart failure models. These antagomiRs work by inhibiting the interaction of miR-21 with its target mRNA, thereby reducing the pathogenic activity of the miRNA. In a clinical context, antagomiRs have great potential, but the main challenges are ensuring specific delivery to the target tissue and minimizing systemic side effects (Cavarretta and Condorelli, 2015).

#### 2.3.2 miRNA mimics

In contrast, miRNA mimics aim to increase the expression of protective miRNAs, such as miR-126 which plays a role in angiogenesis and repairing vascular damage. miRNA mimics mimic the structure of natural miRNAs, thus activating signaling pathways associated with protective functions. In ischemia models, miRNA mimics for miR-126 have shown promising results in improving vascular function and promoting new blood vessel formation (Mierzejewski et al., 2023). However, although this great potential has been identified, there are still significant challenges in translating miRNA-based therapies into clinical practice. One of the biggest challenges is the specific and efficient delivery of antagomiRs or miRNA mimics to target tissues, given that systemic distribution may cause unwanted side effects in other tissues (Chen et al., 2015). The use of nanoparticle technology or extracellular vesicles for targeted delivery has been proposed as a potential solution to this problem. In addition, the stability of miRNAs in the body is also a major concern. Since miRNAs are easily degraded by nuclease enzymes in the plasma, more efficient strategies to protect miRNAs in circulation need to be developed (Cheng et al., 2023). What’s more, since one miRNA can regulate multiple gene targets, there is a risk of pleiotropic effects that should be carefully considered before clinical app lication. Growing technological advances, such as the development of CRISPR-Cas9-based RNA editing and chemical modification of miRNAs to improve stability, give new hope in overcoming these challenges (Afolabi et al., 2021). In addition, the combined use of miRNA-based therapies with conventional drugs offers a more comprehensive approach in treating complex cardiovascular diseases. In the future, miRNAs may become the cornerstone for personalized medicine strategies, where a patient’s miRNA expression pattern is used to determine the most effective and individualized therapy (Zhou X. L. et al., 2018). With a better understanding of the role of miRNAs in cardiovascular pathophysiology, miRNA-based therapies have the potential to be a major breakthrough in the management of cardiovascular disease.

## 3 Flavonoids: cardioprotective agents and their molecular targets

### 3.1 Chemical structure and classification of flavonoids

Flavonoids are a large group of polyphenolic compounds consisting of an aromatic ring-shaped basic structure connected by an oxygen heterocyclic ring. Flavonoids can be found abundantly in plant foods, such as fruits, vegetables, tea, chocolate, and fermented beverages such as red wine (Panche et al., 2016). The diverse chemical structures of flavonoids give them unique bioactivity properties (Dias et al., 2021). In general, flavonoids can be classified into several major subgroups:

#### 3.1.1 Flavonols

Examples of flavonols include quercetin, kaempferol, and myricetin. Flavonols are characterized by the presence of a hydroxyl group at position 3 on the C ring which contributes greatly to their antioxidant activity. Flavonols are well known for their ability to ward off free radicals as well as improve endothelial function through vasodilation (Chagas et al., 2022).

#### 3.1.2 Flavones

Luteolin and apigenin are two flavones found in various fruits and vegetables. Flavones have the basic structure of flavonoids without a hydroxyl group at position 3, and their activity focuses on their anti-inflammatory properties as well as their ability to inhibit abnormal cell proliferation, which plays an important role in protection against vascular disease (Chagas et al., 2022).

#### 3.1.3 Isoflavones

Isoflavones, such as genistein and daidzein found in soybeans, have a unique structure in which the B ring is connected at position 3 of the C ring. Isoflavones are phytoestrogenic, which means they can bind to estrogen receptors and mimic the effects of the hormone estrogen in the body, which provides protection against cardiovascular disease, especially in postmenopausal women (Sathyapalan et al., 2018).

#### 3.1.4 Flavanones

Naringenin and hesperidin, flavanones predominantly found in citrus fruits, have a more saturated C-ring structure than flavones or flavonols. These flavanones have antioxidant, anti-inflammatory effects, and can improve the lipid profile by reducing LDL cholesterol levels in the body (Barreca et al., 2020; Peterson et al., 2006).

#### 3.1.5 Anthocyanins

Anthocyanins, which give fruits like blueberries, grapes, and strawberries their red, blue, or purple color, are flavonoids that have strong antioxidant properties. Cyanidin and delphinidin are examples of anthocyanins that significantly play a role in protecting the heart by reducing oxidative stress and preventing tissue damage from free radicals (Najjar and Feresin, 2021).

Structural Attributes Associated with Biological Activity The chemical structure of flavonoids, especially the number and position of hydroxyl groups, largely determines their bioactivity. The hydroxyl groups on the aromatic ring provide the ability to donate electrons, thus allowing flavonoids to function as effective antioxidants. The lipophilic nature of flavonoids allows them to interact with cell membranes and intracellular signaling pathways, which enhances the role of flavonoids in the modulation of cell responses to oxidative stress and inflammation (Kapral-Piotrowska et al., 2023; Oteiza et al., 2005).

### 3.2 Mechanisms of flavonoid cardioprotective action

**FIGURE 1:**
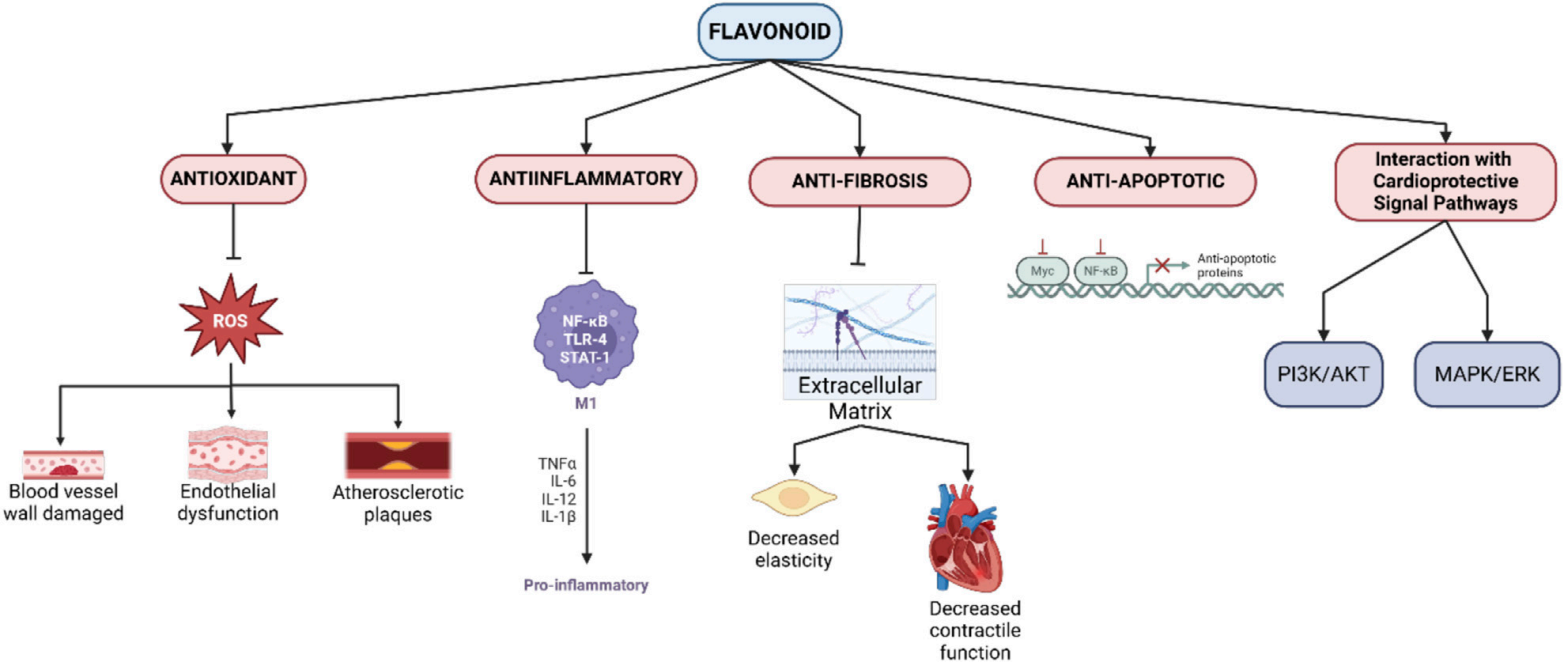
Mechanisms of flavonoid cardioprotective action.

Flavonoids offer multifactorial mechanisms to protect the heart and blood vessels from various types of damage, including those caused by oxidative stress, inflammation, apoptosis, and fibrosis (Figure 1). Each of these mechanisms plays an important role in reducing the risk of developing cardiovascular diseases such as atherosclerosis, hypertension, myocardial infarction, and heart failure (Khan et al., 2021).

#### 3.2.1 Antioxidant activity

One of the main mechanisms of flavonoids is their powerful antioxidant properties. Free radicals, especially reactive oxygen species (ROS), can cause significant damage to the blood vessel wall, promote endothelial dysfunction, and trigger the development of atherosclerotic plaques. Flavonoids such as quercetin, epicatechin, and anthocyanins are able to neutralize ROS and protect endothelial cells from oxidative damage. In addition, flavonoids increase the activity of endogenous antioxidant enzymes such as superoxide dismutase (SOD), catalase, and glutathione peroxidase, strengthening the body’s antioxidant defenses and reducing the risk of systemic inflammation (Ciumărnean et al., 2020).

#### 3.2.2 Anti-inflammatory activity

Chronic inflammation is a major factor in the pathogenesis of cardiovascular diseases, especially atherosclerosis. Flavonoids exhibit anti-inflammatory properties by suppressing the NF-κB pathway, a transcription factor that regulates the production of pro-inflammatory cytokines such as TNF-α, IL-1β, and IL-6. Flavonoids also inhibit the expression of adhesion molecules such as ICAM-1 and VCAM-1, which are responsible for the recruitment of leukocytes to the blood vessel wall. By reducing inflammation, flavonoids not only slow the progression of atherosclerotic plaques, but also reduce the risk of acute cardiovascular events such as heart attacks (Maleki et al., 2019; Serafini et al., 2010).

#### 3.2.3 Anti-apoptotic activity

Damaged myocardial cells, especially after myocardial infarction or ischemia, are prone to apoptosis, which exacerbates heart tissue damage. Flavonoids, such as resveratrol and genistein, have been shown to prevent apoptosis by modulating cellular signaling pathways involving Bcl-2 (anti-apoptotic) and Bax (pro-apoptotic) proteins, as well as inhibiting caspase-3 activation. Through the PI3K/Akt pathway, flavonoids promote myocardial cell survival by increasing resistance to oxidative stress and apoptosis, which is important for maintaining cardiac function (Jia et al., 2020; Khan et al., 2021).

#### 3.2.4 Anti-fibrosis activity

Cardiac fibrosis is a condition in which there is excessive accumulation of extracellular matrix in the heart, leading to decreased elasticity and contractile function of the heart. Flavonoids, specifically naringenin and hesperidin, are able to inhibit the TGF-β pathway, which is known to be a key regulator of fibroblast activation and collagen production. TGF-β inhibition prevents pathological remodeling of cardiac tissue, reduces fibrosis, and maintains post-injury cardiac function (Vistnes, 2024; Parichatikanond et al., 2020).

#### 3.2.5 Interaction with cardioprotective signal pathways

Flavonoids can also affect various signaling pathways that are important for the regulation of cardiovascular function. The PI3K/Akt pathway, for example, is activated by flavonoids such as quercetin and epicatechin, which play important roles in the promotion of cell survival, proliferation, and angiogenesis. In addition, flavonoids can suppress the MAPK/ERK pathway associated with inflammation and abnormal cell proliferation, providing protection against the development of vascular diseases such as hypertension and atherosclerosis (Suhail et al., 2023; Zhang et al., 2018).

### 3.3 Pharmacokinetics and bioavailability of flavonoids

Flavonoids present notable challenges in therapeutic applications due to their low bioavailability, defined as the proportion of active flavonoid compounds that reach systemic circulation following oral administration. Factors influencing flavonoid bioavailability include their chemical structure, metabolic pathways, and mechanisms of delivery within the body (Thilakarathna and Rupasinghe, 2013).

#### 3.3.1 Absorption

Flavonoids are generally ingested as glycosides, bound to sugar moieties, and must undergo hydrolysis in the small intestine via enzymes such as β-glucosidase or through gut microbiota activity to be converted into free aglycone forms suitable for absorption. Post-hydrolysis, flavonoid aglycones may be absorbed either through passive diffusion or via active transporters, including GLUT2 and SGLT1, present in intestinal enterocytes. However, absorption efficiency varies widely, contingent upon each flavonoid’s specific structural characteristics (Zhao et al., 2019; Hollman, 2004).

#### 3.3.2 Distribution

Upon absorption, flavonoids experience first-pass metabolism in the liver, undergoing conjugation via glucuronidation, sulfation, or methylation by Phase II enzymes. The resulting flavonoid metabolites are then disseminated across various tissues, including the cardiovascular system and central nervous system, where they may interact with molecular targets. Although the biological activity of flavonoid metabolites is typically lower than that of their parent compounds, emerging evidence suggests that these metabolites retain measurable biological activity, albeit at reduced potency (Mansuri et al., 2014).

#### 3.3.3 Metabolism

Flavonoids are extensively metabolized within the liver and intestine, primarily through Phase II enzymes, such as UDP-glucuronosyltransferase (UGT) and sulfotransferase, resulting in glucuronide and sulfate conjugates that circulate systemically. While these polar metabolites are more readily excreted, they nonetheless maintain significant biological properties, contributing to the overall pharmacodynamic profile of flavonoids (Boronat et al., 2021; Yang et al., 2017).

#### 3.3.4 Excretion

The primary excretion pathways for flavonoids and their metabolites include renal (urine) and biliary (bile) routes. Biliary excretion permits flavonoids to undergo enterohepatic circulation, which may prolong their plasma half-life and enhance clinical efficacy. Nonetheless, the majority of flavonoids are excreted in glucuronide and sulfate-conjugated forms through renal pathways (Hollman, 2004).

### 3.4 Factors influencing flavonoid bioavailability

The bioavailability of flavonoids—defined as the fraction of ingested compounds that reach systemic circulation in their active form—presents a significant challenge in the clinical application of flavonoids as therapeutic agents. Despite their promising bioactive properties, flavonoids frequently exhibit low bioavailability due to multiple factors, including chemical structure, dietary interactions, and extensive metabolism in the gastrointestinal tract and liver (Thilakarathna and Rupasinghe, 2013; Zhao et al., 2019). A detailed analysis of these influencing factors and approaches to optimize bioavailability is presented below.

#### 3.4.1 Chemical structure of flavonoids

The intrinsic chemical structure of flavonoids substantially determines their bioavailability. Factors such as the presence, number, and positioning of hydroxyl groups, saturation of the C-ring, and glycosylation status (the presence of sugar moieties) critically affect solubility and the capacity of flavonoids to cross cellular barriers in the intestine (Wang et al., 2020; Fang et al., 2017).

Hydroxyl Group Count and Positioning: Hydroxyl (-OH) groups attached to the aromatic rings of flavonoids influence their antioxidant properties, solubility, and polarity. Flavonoids with numerous hydroxyl groups generally exhibit enhanced water solubility; however, they may exhibit reduced membrane permeability, limiting their absorption. For instance, quercetin, a highly hydroxylated flavonol, displays lower bioavailability than flavonoids with fewer hydroxyl groups, such as the flavanone naringenin (Mohammadi et al., 2024).

Degree of Saturation in the C-Ring: The saturation of the C-ring impacts the rigidity of the flavonoid structure, affecting bioavailability (Wang et al., 2017). Flavonoids with a saturated C-ring (e.g., flavanones) display greater structural flexibility, facilitating passive membrane diffusion and cellular absorption. In contrast, flavonoids containing a double bond in the C-ring (e.g., flavones) are less flexible, often resulting in lower lipid solubility and decreased intestinal absorption (Hollman, 2004).

Glycosylation vs. Aglycone Forms: Flavonoids in their glycosylated forms, prevalent in dietary sources, require enzymatic hydrolysis in the gut to release the more absorbable aglycone form (Yang et al., 2020). Aglycones generally demonstrate superior bioavailability, as their increased lipophilicity facilitates passive diffusion across cell membranes (Graefe et al., 2001). Conversely, glycosylated flavonoids, such as rutin (quercetin glycoside), exhibit reduced absorption due to the enzymatic processes required for conversion into their bioactive aglycone counterparts (Yang et al., 2020).

##### 3.4.1.1 Gut microbiota

The composition and metabolic capacity of gut microbiota play an essential role in modulating flavonoid bioavailability. Intestinal microorganisms have the capacity to biotransform flavonoids into active metabolites that are more readily absorbed or, alternatively, into less bioactive forms (Pan et al., 2023).

Microbial Biotransformation of Flavonoids: Specific gut microbes hydrolyze flavonoid glycosides, yielding absorbable aglycone forms. Moreover, microbial metabolism produces metabolites, such as small phenols and aromatic acids, that can enter systemic circulation. For instance, microbial conversion of the isoflavone genistein results in metabolites with augmented estrogenic activity, which may confer additional protective effects against cardiovascular disease (Pan et al., 2023; Baky et al., 2021).

Interindividual Microbiota Variability: The gut microbiota composition exhibits substantial interindividual variability, influencing the efficiency of flavonoid metabolism. Such differences may account for the observed variability in health outcomes associated with flavonoid consumption across populations, despite similar dosages (Pan et al., 2023).

##### 3.4.1.2 Dietary interactions

Interactions between flavonoids and other dietary components significantly impact their bioavailability. Co-ingestion with specific nutrients, particularly fats, has been shown to enhance flavonoid absorption, as fats promote dissolution of lipophilic compounds and slow gastric emptying, thereby extending the absorption window (Hollman, 2004).

Lipid Influence on Flavonoid Bioavailability: Studies suggest that co-consumption of lipophilic flavonoids, such as naringenin and quercetin, with dietary fats enhances bioavailability. Lipid micelles formed during digestion can dissolve flavonoids, facilitating their uptake across intestinal epithelial cells (Thilakarathna and Rupasinghe, 2013).

Polyphenol-Polyphenol Competition: Flavonoids may compete with other polyphenols for intestinal transporters, which may reduce bioavailability. Consequently, the overall dietary composition and the interaction of multiple polyphenolic compounds play a crucial role in modulating the bioavailability of individual flavonoids (Thilakarathna and Rupasinghe, 2013).

### 3.5 Strategies to enhance flavonoid efficacy and bioavailability

**FIGURE 2:**
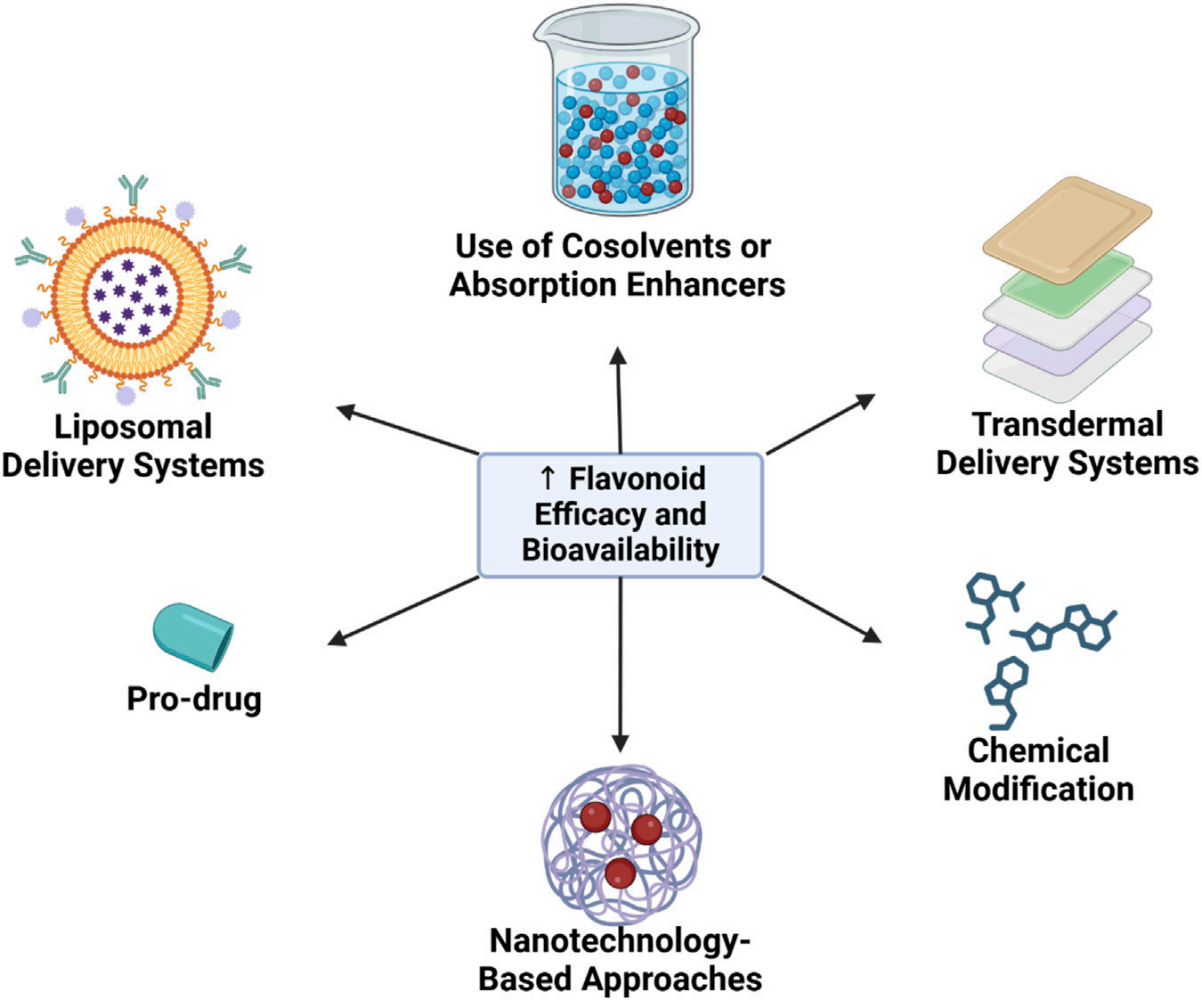
Strategies to enhance flavonoid efficacy and bioavailability.

Given the considerable challenges in flavonoid bioavailability, several innovative strategies have been developed to improve both stability and therapeutic efficacy. The following are some of the most promising approaches for enhancing flavonoid bioavailability (Figure 2).

#### 3.5.1 Nanotechnology-based approaches

Nanotechnology has emerged as a pivotal approach for enhancing flavonoid stability and absorption. Encapsulation of flavonoids within nanoparticles or nanocapsules has demonstrated significant potential in overcoming barriers to bioavailability.

Nanoparticle Encapsulation: Encapsulation of flavonoids in lipid or polymer-based nanoparticles shields them from enzymatic degradation within the gastrointestinal tract, prolonging intestinal residence time and enhancing absorption. Nanoparticles also allow for targeted delivery to specific tissues, such as the myocardium or vascular endothelium, via endocytotic mechanisms (Syahputra et al., 2024).

Increased Solubility through Particle Size Reduction: Nanoparticles, with their reduced particle size, exhibit improved solubility in aqueous environments, enhancing diffusion across the intestinal barrier. For instance, quercetin nanoparticles have demonstrated markedly enhanced bioavailability compared to conventional forms (Syahputra et al., 2024).

#### 3.5.2 Pro-drug and chemical modification strategies

Structural modification of flavonoids offers a promising avenue for enhancing bioavailability. Pro-drug approaches and chemical modifications are utilized to optimize solubility and metabolic stability.

Pro-drug Design: Pro-drugs are chemically modified derivatives that improve solubility or stability but are reconverted to active forms upon metabolism. For example, flavonoids may be modified to increase hydrophilicity, improving absorption, and subsequently revert to the active aglycone form within target tissues (Lee et al., 2016; Halbwirth, 2010).

Hydroxyl Group Modifications: Modifications such as methylation or addition of fatty acids to hydroxyl groups increase lipophilicity, enhancing flavonoid permeability through the lipid membranes of intestinal cells. Such modifications also reduce enzymatic degradation rates, thereby prolonging the metabolic half-life of flavonoids within the body (Lee et al., 2016).

#### 3.5.3 Liposomal delivery systems

Liposomes, spherical phospholipid bilayers capable of encapsulating bioactive compounds, serve as effective carriers for enhancing flavonoid bioavailability. Liposomal encapsulation provides several key benefits:

Protection from Enzymatic Degradation: Liposomes protect flavonoids from degradation within the gastrointestinal tract, enabling a greater quantity of the active compound to reach systemic circulation (Huang et al., 2017).

Targeted Drug Delivery: Liposomes can be engineered to bind specifically to certain cell types or tissues, facilitating directed delivery of flavonoids to the myocardium, vasculature, or diseased tissues. This targeted approach enhances the therapeutic efficacy of flavonoids while minimizing systemic adverse effects (Huang et al., 2017).

#### 3.5.4 Use of cosolvents or absorption enhancers

The use of cosolvents or absorption enhancers is one strategy to improve the solubility and absorption of flavonoids in the body. Cosolvents, such as ethanol, propylene glycol, and surfactants, can enhance the solubility of flavonoids within the digestive system, thereby improving bioavailability. Additionally, some absorption enhancers, like piperine—a compound found in black pepper—have shown potential in enhancing the bioavailability of flavonoids by inhibiting metabolic enzymes like UGT, which are involved in flavonoid conjugation (He et al., 2022).

#### 3.5.5 Transdermal delivery systems

The transdermal delivery approach offers an alternative for addressing the bioavailability challenges associated with oral flavonoid absorption. By using transdermal patches or gels containing flavonoids, these compounds can be absorbed directly into the bloodstream without first-pass metabolism in the liver. This method has the potential to significantly improve flavonoid bioavailability, providing a more effective option for therapeutic applications (Costa et al., 2021; Cheng et al., 2020).

## 4 Flavonoids and miRNA modulation: mechanistic insights

The interaction between flavonoids and microRNA (miRNA) within the context of cardiovascular health represents a rapidly advancing area of research. Flavonoids, a large group of polyphenolic compounds with diverse biological activities, have been shown to modulate miRNA expression through both direct and epigenetic regulation. Given that miRNAs are key regulators in the development and pathogenesis of cardiovascular diseases (CVD), such as hypertension, atherosclerosis, and heart failure, understanding how flavonoids influence miRNA opens new opportunities for molecular therapies in heart disease treatment (Syahputra et al., 2024).

### 4.1 Mechanisms of flavonoid-miRNA interaction

Flavonoids can modulate miRNA expression via several mechanisms, either directly by interacting with signaling molecules or indirectly through epigenetic regulation. These mechanisms provide a foundation for developing more effective flavonoid-based therapies.

#### 4.1.1 Direct modulation of miRNA expression

Certain flavonoids can directly affect miRNA expression by modulating the activity of transcription factors or proteins that control the transcription of specific miRNAs. For instance, resveratrol activates signaling pathways involving transcription factors SIRT1 and FOXO3, which increase the expression of miR-126, thereby enhancing angiogenesis and improving endothelial function (Wiciński et al., 2023). Meanwhile, quercetin inhibits miR-21 expression, helping to reduce cardiac fibrosis and inflammation (Zhang et al., 2021).

#### 4.1.2 Epigenetic regulation

Flavonoids also play a role in regulating miRNA expression via epigenetic mechanisms, such as histone modifications, DNA methylation, and the regulation of epigenetic enzymes (e.g., HDAC and DNMT). For example, genistein inhibits DNMT, promoting the expression of the anti-fibrotic miRNA miR-29, while EGCG (epigallocatechin gallate) inhibits HDAC, thereby upregulating miR-15 expression, which prevents apoptosis (Wang et al., 2021; Zhu et al., 2018; Hirata et al., 2013).

### 4.2 Impact of flavonoids on cardiovascular-specific miRNA

Preclinical and clinical studies demonstrate that flavonoids exert a significant impact on miRNA associated with cardiovascular health. The following are specific examples of flavonoid effects on miRNA expression:

Quercetin and miR-21: Quercetin downregulates miR-21 expression, which reduces fibroblast activation and excessive collagen production in the heart. This reduction helps prevent post-myocardial infarction fibrosis and atherosclerotic plaque formation (Dostal and Modriansky, 2019).

Resveratrol and miR-34a: Resveratrol downregulates miR-34a expression, reducing apoptosis in myocardial cells and enhancing recovery post-ischemic injury, thereby helping to prevent excessive scarring (Pan et al., 2022).

Genistein and miR-29: Genistein upregulates miR-29 expression, reducing excessive collagen synthesis associated with cardiac fibrosis, thereby preventing pathological remodeling in hypertrophic heart conditions (Wang et al., 2021).

### 4.3 Potential signaling pathways involved

Certain molecular signaling pathways involved in miRNA modulation by flavonoids play a key role in cardiovascular health. These pathways are not only relevant for maintaining normal heart and vascular function but also in repairing damage caused by cardiovascular diseases:

PI3K/AKT Pathway: This pathway is essential for cell survival and vascular growth. Flavonoids like epicatechin and quercetin are known to activate this pathway, enhancing the expression of miR-126 to promote endothelial cell survival, stimulate angiogenesis, and reduce apoptosis (Cai et al., 2024; Rascio et al., 2021).

NF-κB Pathway: This pathway regulates inflammatory responses. Flavonoids like kaempferol and apigenin inhibit NF-κB, reducing the expression of pro-inflammatory miRNAs, such as miR-155, which helps lower the risk of vascular inflammation and atherosclerosis (Li and Zhang, 2023; Cao et al., 2016).

TGF-β Pathway: This pathway is associated with fibrosis regulation. Flavonoids like hesperidin and naringenin suppress TGF-β pathway activity by reducing miR-21 expression, which is linked to cardiac tissue fibrosis. The downregulation of miR-21 through TGF-β inhibition contributes to reduced cardiac fibrosis and vascular remodeling, thus preventing cardiac dysfunction and heart failure (Xu et al., 2021).

#### 4.3.1 Impact on endothelial function, smooth muscle cell proliferation, and vascular inflammation

miRNA modulation by flavonoids directly influences various processes critical to cardiovascular health:

Improvement of Endothelial Function: Endothelial dysfunction is an initial mechanism in cardiovascular disease development. By increasing the expression of miRNAs such as miR-126, flavonoids can enhance endothelial function by stimulating nitric oxide (NO) production and reducing vascular permeability. This is crucial for preventing atherosclerotic plaque formation and maintaining vascular elasticity (Liu et al., 2018).

Inhibition of Smooth Muscle Cell Proliferation: Vascular smooth muscle cell (VSMC) proliferation contributes to arterial lumen narrowing and atherosclerotic plaque formation. Flavonoids, such as quercetin and kaempferol, reduce this proliferation by modulating miRNAs like miR-21 and miR-145, thereby inhibiting VSMC proliferation and preventing arterial stenosis (Kang and Hata, 2012).

Reduction of Vascular Inflammation: Flavonoids decrease vascular inflammation by suppressing the expression of pro-inflammatory miRNAs, such as miR-155, which reduces immune cell infiltration into atherosclerotic plaques (Bruen et al., 2019). By mitigating inflammation, flavonoids help prevent atherosclerosis progression and lower the risk of acute cardiovascular events, such as myocardial infarction or stroke (Al-Khayri et al., 2022; Cao et al., 2016).

## 5 Preclinical and clinical evidence

The modulation of microRNAs (miRNAs) by flavonoids within the context of cardiovascular disease (CVD) represents an emerging area of research, with growing evidence suggesting beneficial effects of flavonoids on cardiovascular health via miRNA regulation. From preclinical studies to ongoing clinical trials, there is significant potential to integrate flavonoids as a therapeutic option for CVD through their impact on miRNAs (Kura et al., 2019). This section will explore preclinical and clinical evidence on flavonoid-miRNA interactions in CVD treatment, as well as opportunities for the use of flavonoids in personalized medicine.

### 5.1 Preclinical studies: flavonoid-miRNA interaction in cardiovascular models

Preclinical studies are essential for understanding the mechanisms behind the therapeutic effects of flavonoids through miRNA modulation. Research is conducted both *in vitro*, using cell cultures, and *in vivo*, using animal models—primarily rats and mice induced with cardiovascular conditions, such as hypertension, atherosclerosis, and myocardial infarction.

#### 5.1.1 *In vitro* studies

*In vitro* research often employs endothelial cells, vascular smooth muscle cells, or cardiac fibroblasts to evaluate how flavonoids affect the expression of miRNAs related to CVD pathogenesis. Key findings from *in vitro* studies include:

*Quercetin and miR-21*: Quercetin, a flavonoid commonly found in vegetables and fruits, has been shown to reduce miR-21 expression in cardiac fibroblasts. miR-21 is a pro-fibrotic miRNA that plays a significant role in fibroblast activation and excessive collagen formation in cardiac tissue. By lowering miR-21 expression, quercetin can inhibit fibrosis processes and prevent cardiac remodeling caused by myocardial infarction (Wang et al., 2024).

*Epigallocatechin Gallate (EGCG) and miR-126*: In human endothelial cell cultures, EGCG—a key flavonoid in green tea—has been shown to increase miR-126 expression, a miRNA crucial for angiogenesis and endothelial regeneration. This increase in miR-126 contributes to improved vascular function, particularly in conditions of endothelial dysfunction resulting from diabetes or hypertension (Arderiu et al., 2022; Zhang et al., 2022a).

#### 5.1.2 *In vivo* studies

Animal models, such as hypertensive or atherosclerotic rats and mice, are used to understand flavonoid effects on CVD within a complex organismal context. Flavonoids are administered via dietary supplements or injections to assess their systemic effects on cardiac and vascular tissues. Significant findings from *in vivo* studies include:

*Resveratrol and miR-34a*: In hypertensive rat models, resveratrol, a flavonoid found in red grapes, has been shown to reduce miR-34a expression, a miRNA associated with cardiac aging and cellular apoptosis. Lower miR-34a levels correlate with decreased myocardial cell apoptosis, prolonged cardiac cell survival, and enhanced cardiac resistance to oxidative stress damage (Giordo et al., 2022; Bonnefont-Rousselot, 2016).

*Genistein and miR-29*: In myocardial infarction rat models, genistein, an isoflavone found in soybeans, has been shown to increase miR-29 expression. miR-29 inhibits excessive collagen accumulation, prevents cardiac fibrosis, and promotes myocardial tissue repair after injury. This research suggests genistein may reduce cardiac remodeling following infarction (Poasakate et al., 2021; Bitto et al., 2008).

#### 5.1.3 Preclinical findings on CVD improvement through miRNA modulation by flavonoids

Preclinical studies, including cell cultures and animal models, demonstrate that flavonoids can target specific miRNAs involved in the pathogenic pathways of CVD. Key findings from these studies include:

Reduction of Cardiac Fibrosis: Quercetin and naringenin, two major flavonoids found in citrus fruits, reduce the expression of miR-21, which plays a role in cardiac fibrosis and pathological remodeling. This effect is particularly important in heart failure models, where fibrosis reduces cardiac elasticity and contractile ability (Zhang et al., 2021).

Improvement of Endothelial Function: Flavonoids such as epicatechin and resveratrol have been shown to increase the expression of miR-126, promoting endothelial regeneration, enhancing angiogenesis, and improving vascular dysfunction, particularly in myocardial ischemia and hypertension models (Sanguineti et al., 2021).

Reduction of Vascular Inflammation: Flavonoids like kaempferol and apigenin inhibit the expression of miR-155, a pro-inflammatory miRNA involved in immune cell infiltration into atherosclerotic plaques. This reduces vascular inflammation, slows the progression of atherosclerosis, and lowers the risk of acute cardiovascular events, such as heart attacks and strokes (Al-Khayri et al., 2022; Bruen et al., 2019; Cao et al., 2016).

### 5.2 Clinical studies and trials

Following promising results in preclinical research, some flavonoids have advanced to clinical trials to evaluate their effects on miRNA expression and cardiovascular health outcomes in humans. While preclinical findings are positive, the transition from laboratory to clinical application often faces challenges, particularly concerning bioavailability and interindividual variability.

#### 5.2.1 Ongoing clinical trials

Several clinical trials are currently investigating how flavonoids, such as resveratrol, quercetin, and epicatechin, affect miRNA expression related to cardiovascular health. Key studies include:

*Resveratrol and Endothelial Function*: Clinical trials are assessing the effects of resveratrol supplementation on the expression of miRNAs, such as miR-126, to determine if the positive effects observed in animal models are replicable in humans. The primary focus of these studies is to improve endothelial dysfunction in patients with hypertension or atherosclerosis by enhancing miRNA expression related to vascular regeneration (Brown et al., 2024; McCubrey et al., 2017).

*Quercetin and Vascular Inflammation*: Another study is evaluating the impact of quercetin on inflammatory biomarkers and miR-155 expression in patients with atherosclerosis. The goal is to determine whether quercetin can suppress vascular inflammation and improve lipid profile and blood pressure, which are crucial for coronary artery disease prevention (Ou et al., 2020).

#### 5.2.2 Challenges and opportunities in translating preclinical evidence to clinical practice

Although preclinical evidence suggests a high potential for flavonoids in miRNA modulation for CVD management, several challenges remain in translating these findings to clinical practice. Key challenges include:

*Low Bioavailability*: Flavonoids often have low bioavailability in humans due to poor absorption and rapid metabolism, which limits the active concentration of flavonoids in systemic circulation and reduces their effectiveness in miRNA modulation (Thilakarathna and Rupasinghe, 2013). Clinical studies need to address optimal delivery methods and dosing to overcome this challenge.

*Interindividual Response Variability*: Genetic factors, diet, and gut microbiota composition can cause significant variation in response to flavonoid supplementation. This variability can impact clinical trial outcomes and pose difficulties in designing broadly applicable therapeutic protocols (Xiong et al., 2023).

*Therapy Duration*: Most clinical trials are short-term, while miRNA modulation effects may require a longer duration to be observable. Long-term studies are needed to evaluate the safety and efficacy of flavonoids in miRNA modulation and in preventing cardiovascular disease progression (Seyhan, 2024).

### 5.3 Potential of flavonoids as miRNA modulators in personalized medicine

In personalized medicine, an approach tailored to individual genetic and molecular profiles is increasingly important. Flavonoid interactions with specific miRNAs have potential as a basis for developing personalized therapies for CVD, where patients with certain miRNA profiles may receive the most appropriate flavonoid intervention.

#### 5.3.1 Role of flavonoid-miRNA interactions in precision medicine

miRNA modulation by flavonoids creates opportunities for more specific interventions based on individual miRNA profiles. In the context of precision medicine, flavonoid use can be optimized by tailoring therapies to target specific miRNAs known to be associated with CVD in each patient. For example:

*Patients with Cardiac Fibrosis*: Patients with elevated expression of miR-21, which contributes to cardiac fibrosis, may benefit from flavonoid therapies such as quercetin, known to inhibit miR-21, thereby reducing fibrosis risk and improving cardiac function (Xu et al., 2021).

*Patients with Atherosclerosis*: Patients with a miRNA profile showing increased miR-155 expression may be treated with flavonoid supplements such as kaempferol, which reduces miR-155, decreasing vascular inflammation and slowing plaque progression (Yao et al., 2020).

#### 5.3.2 miRNA profile-based intervention customization

A miRNA profile-based approach can help physicians select the most effective flavonoids for individual therapy. For instance, a patient’s miRNA profile could be identified through blood tests to determine the dominant miRNA expressions, and based on this information, the most effective flavonoid to target these miRNAs can be chosen. This enables more personalized and efficient treatment, focused on reducing side effect risks and improving clinical outcomes.

## 6 Challenges and future perspectives

Although research on flavonoids and miRNA modulation in cardiovascular disease (CVD) is advancing, several key challenges must still be addressed. From limitations in understanding the specific interactions between flavonoids and miRNAs to low bioavailability, numerous barriers hinder the translation of preclinical findings into clinical applications. Furthermore, with advances in technology and research in molecular biology, new opportunities are emerging to address these challenges and expand the potential of flavonoids as therapeutic agents in miRNA modulation.

### 6.1 Challenges in flavonoid and miRNA research

Research on the interaction between flavonoids and miRNAs is still in its early stages, with many aspects not fully understood. Current key challenges in this research include.

#### 6.1.1 Limitations in understanding the specificity of flavonoid-miRNA interactions

A major challenge in flavonoid and miRNA research is the lack of a clear understanding of the specific interactions between flavonoid compounds and particular miRNAs. While some flavonoids have shown potential in modulating the expression of certain miRNAs, the underlying mechanisms often involve complex and multifactorial signaling pathways. Flavonoids, being polyphenolic compounds, tend to have multifactorial effects and may influence multiple biological targets simultaneously. Precisely identifying how flavonoids affect specific miRNAs in different cardiovascular contexts requires further investigation. Additionally, many miRNAs target a wide array of genes, so modulating a single miRNA through flavonoids may have widespread effects on numerous biological pathways. Identifying specific miRNAs that flavonoids can modulate, as well as the gene targets associated with these miRNAs, remains a significant challenge that requires advanced technology and a deep understanding of molecular biology (Tuli et al., 2023; Adinew et al., 2021).

#### 6.1.2 Issues of bioavailability, dosage, and long-term safety

Low bioavailability is a primary barrier to using flavonoids as a therapy. Many flavonoids have poor water solubility and are rapidly metabolized in the gastrointestinal tract and liver (first-pass effect), leading to very low concentrations in systemic circulation. Additionally, there is limited development of methods to effectively increase flavonoid concentrations in target tissues without increasing toxicity.

Determining the correct dosage is also challenging, as preclinical studies often employ flavonoid doses that are much higher than what can be realistically administered to humans. Lower doses in clinical studies may not yield the same effects observed in animal models.

Long-term safety is another concern, especially since flavonoids are naturally present in the human diet. However, consuming high doses of flavonoids over extended periods may have side effects that are not yet fully explored. Long-term studies are thus needed to evaluate the safety of high-dose flavonoid supplementation in humans (Williamson et al., 2018; Thilakarathna and Rupasinghe, 2013).

### 6.2 Advances in technology and research opportunities

Despite these challenges, advances in molecular technology and synthetic biology are opening new opportunities for studying flavonoid-miRNA interactions and developing more effective, targeted therapies.

#### 6.2.1 miRNA expression profiling and flavonoid interaction technologies

High-throughput sequencing technologies, such as RNA-seq, have enabled large-scale analysis of miRNA expression, providing a deeper understanding of how flavonoids affect miRNA expression systemically. These technologies allow researchers to identify specific miRNAs modulated by flavonoids across various types of cardiovascular cells, including endothelial cells, vascular smooth muscle cells, and cardiac fibroblasts.

CRISPR-based tools also offer opportunities for more precise miRNA manipulation. By using CRISPR systems to edit or delete specific miRNAs, researchers can better understand the role of miRNAs in CVD and how flavonoids influence miRNA expression and function. This offers a chance to develop more targeted flavonoid-based therapies by focusing on highly specific miRNAs (Abkhooie and Saberianpour, 2023; Hoffmann et al., 2019).

#### 6.2.2 Opportunities for synthetic biology approaches

Synthetic biology offers opportunities to optimize flavonoids as miRNA modulators through chemical modification and metabolite engineering. Using synthetic biology techniques, flavonoid compounds can be designed to target specific miRNAs more efficiently. For example, flavonoids can be modified to enhance stability or affinity for cardiac tissues, thus increasing their therapeutic effects (Tariq et al., 2023; Sheng et al., 2020).

Additionally, synthetic flavonoid analogs mimicking the biological properties of natural flavonoids can be optimized to improve bioavailability and miRNA modulation potential. These synthetic compounds can be designed to have better pharmacokinetic profiles than natural flavonoids, enabling the development of more effective therapies for managing CVD.

### 6.3 Future directions

In the long term, flavonoids hold promising prospects as miRNA modulators for the treatment of cardiovascular disease (CVD). With advancing technology and research in this field, several future directions could further explore the role of flavonoids in miRNA modulation.

#### 6.3.1 Development of flavonoid-based drugs for miRNA modulation in CVD

The development of flavonoid-based drugs specifically designed to modulate CVD-related miRNAs will be a critical area moving forward. As our understanding of miRNA-mediated molecular pathways deepens, flavonoids could be optimized to target specific miRNAs associated with cardiac and vascular pathology, such as miR-21 (fibrosis), miR-155 (inflammation), or miR-126 (endothelial function).

This development will involve structural modification of flavonoids to improve the selectivity and potency of miRNA modulation, along with adjustments in dosage and delivery methods to more effectively reach desired target tissues (Sanguineti et al., 2021; Bruen et al., 2019; Zhou S. et al., 2018; Chau et al., 2013).

#### 6.3.2 Combining flavonoids with existing miRNA-based therapies

The potential to combine flavonoids with existing miRNA therapies to achieve synergistic effects should also be explored. miRNA-based therapies, such as antagomiRs (miRNA inhibitors) or miRNA mimics, have shown great promise in regulating gene expression linked to CVD. Combining these therapies with flavonoids could result in more robust and specific therapeutic effects, optimizing therapeutic benefits while minimizing side effects.

For example, combining quercetin with antagomiR for miR-21 could result in more effective inhibition of cardiac fibrosis, while combining flavonoids like resveratrol with miRNA mimics for miR-126 may enhance angiogenesis in ischemic conditions (Tuli et al., 2023; Sanguineti et al., 2021).

#### 6.3.3 Personalization of flavonoid-based therapy with miRNA profiling

Within the framework of precision medicine, flavonoid-based interventions could be tailored to an individual’s miRNA profile. Genetic and molecular testing could be used to identify patients' miRNA expression patterns, allowing for the selection of the most effective flavonoids for modulating specific miRNAs. This approach not only enhances therapeutic efficacy but also reduces the risk of unwanted side effects (Zhong et al., 2016).

With the continued advancement of miRNA profiling and the understanding of flavonoid interactions with molecular pathways, this personalized approach could pave the way for more targeted and effective treatments for cardiovascular disease.

## 7 Conclusion

MicroRNAs (miRNAs) play a pivotal role in cardiovascular dysfunction by regulating molecular pathways that drive inflammation, apoptosis, and fibrosis. Flavonoids, with their bioactive properties, have shown substantial potential in modulating miRNA expression related to cardiovascular disease, such as miR-21, miR-126, and miR-155, which are involved in cardiac fibrosis, endothelial function, and vascular inflammation. Preclinical studies provide strong evidence that flavonoids can effectively target miRNAs to improve cardiovascular health; however, transitioning these findings to clinical applications will require further research to address challenges such as bioavailability, specificity, and long-term safety. With advancing technologies like miRNA expression profiling and synthetic biology approaches, flavonoids hold potential as an innovative therapeutic strategy for managing cardiovascular disease, and they may also open avenues for the development of personalized treatments based on individual miRNA profiles.
